# P-1338. In vitro activity of ceftazidime-avibactam and comparator agents against Pseudomonas aeruginosa stratified by infection source and ward: ATLAS Global Surveillance Program, 2019-2023

**DOI:** 10.1093/ofid/ofaf695.1526

**Published:** 2026-01-11

**Authors:** Meredith Hackel, Gregory Stone, Katherine Perez, Paurus Irani, Daniel F Sahm

**Affiliations:** IHMA, Schaumburg, IL; Pfizer, Inc., Groton, Connecticut; Pfizer, Inc., Groton, Connecticut; Pfizer United Kingdom, London, England, United Kingdom; IHMA, Schaumburg, IL

## Abstract

**Background:**

The β-lactamase inhibitor, avibactam, has potent inhibitory activity against Class A, Class C, and certain Class D serine β-lactamases. This study evaluated the *in vitro* activity of ceftazidime-avibactam (CZA) and comparators against clinical isolates of *Pseudomonas aeruginosa* collected for the ATLAS global surveillance program in 2019-2023 stratified by source of infection and ward.

**Figure:**
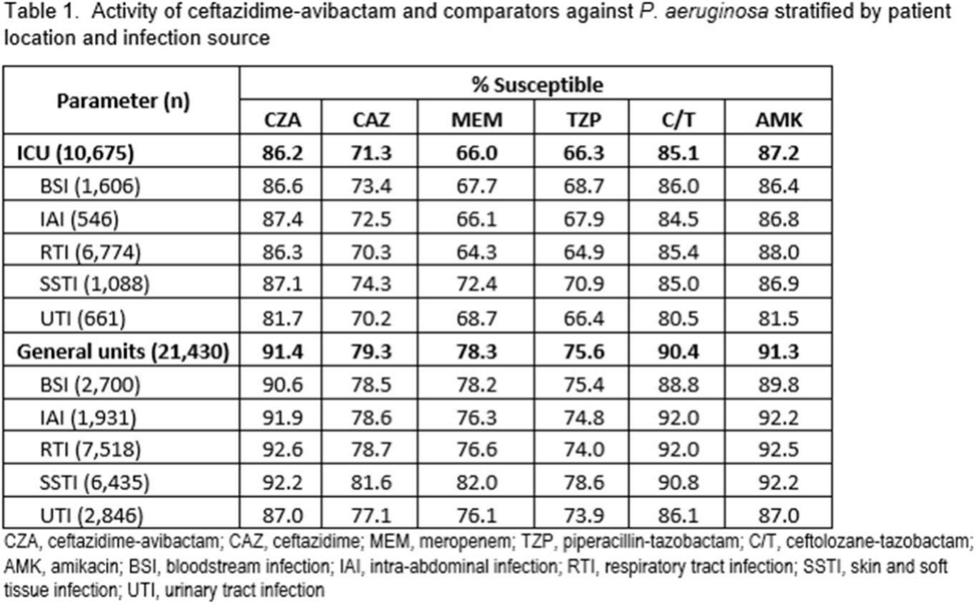

**Methods:**

32,105 isolates of *P. aeruginosa* collected from 264 medical centers in 58 countries as part of the ATLAS program from 2019-2023 (excluding North America) for which the patient location was either a general ward (n=21,430) or an ICU (n=10,675) and infection source was specified were evaluated. Infection sources included BSI (bloodstream infection), IAI (intra-abdominal infection), RTI (respiratory tract infection), SSTI (skin and soft tissue infection) and UTI (urinary tract infection). Susceptibility testing was performed by broth microdilution following the CLSI standard method and analyzed using CLSI 2024 breakpoints.

**Results:**

Results are shown in Table 1. CZA (86.2% S) and amikacin (87.2% S) were the most active agents tested against isolates from ICUs. Against isolates from general wards, CZA (91.4% S) and amikacin (91.3% S) were the most active agents. Activity of CZA ranged from 81.7% S to 87.4% S across infection sources from ICU isolates, and 87.0% S to 92.6% S for isolates from general wards. For all agents tested, %S was higher in general wards compared to ICUs by 4.1 (amikacin) to 12.3 (meropenem) percentage points.

**Conclusion:**

CZA is active against all groupings of *P. aeruginosa* regardless of patient location or infection source. These data suggest CZA remains an excellent therapeutic choice to consider against *P. aeruginosa*.

**Disclosures:**

Katherine Perez, PhD, Pfizer: Stocks/Bonds (Public Company) Paurus Irani, MD, Pfizer, Inc.: Employee|Pfizer, Inc.: Stocks/Bonds (Private Company)

